# Estimating Arterial Cyclic Strain from the Spacing of Endothelial Nuclei

**DOI:** 10.1007/s11340-020-00655-9

**Published:** 2020-09-09

**Authors:** E.M. Rowland, E.L. Bailey, P.D. Weinberg

**Affiliations:** 1 Department of Biomedical Engineering, Imperial College London, London SW7 2AZ, UK

**Keywords:** Arterial strain, Cyclic arterial strain, Corrosion casting, Nuclear spacing, Nearest neighbour analysis, Aorta

## Abstract

**Background:**

The non-uniform distribution of atherosclerosis within the arterial system is widely attributed to variation in haemodynamic wall shear stress. It may also depend on variation in pressure-induced stresses and strains within the arterial wall; these have been less widely investigated, at least in part because of a lack of suitable techniques.

**Objectives:**

Here we show that local arterial strain can be determined from impressions left by endothelial cells on the surface of vascular corrosion casts made at different pressures, even though only one pressure can be examined in each vessel. The pattern of pits in the cast caused by protruding endothelial nuclei was subject to “retro-deformation” to identify the pattern that would have occurred in the absence of applied stresses.

**Methods:**

Retaining the nearest-neighbour pairs found under this condition, changes in nearest-neighbour vectors were calculated for the pattern seen in the cast, and the ratio of mean changes at different pressures determined. This approach removes errors in simple nearest-neighbour analyses caused by the nearest neighbour changing as deformation occurs.

**Results:**

The accuracy, precision and robustness of the approach were validated using simulations. The method was implemented using confocal microscopy of casts of the rabbit aorta made at systolic and diastolic pressures; results agreed well with the ratio of the macroscopic dimensions of the casts.

**Conclusions:**

Applying the new technique to areas around arterial branches could support or refute the hypothesis that the development of atherosclerosis is influenced by mural strain, and the method may be applicable to other tissues.

## Introduction

Atherosclerosis is characterised by the accumulation of lipids, cells and fibrous proteins within the arterial wall. Systemic factors govern its prevalence and severity but they cannot account for the highly non-uniform distribution of lesions within the arterial system. Its predilection for areas of branching and curvature has given rise to the view that haemodynamic stresses play an important role in the pathogenesis of the disease. Low and oscillatory wall shear stress (WSS), the frictional force per unit area between the blood and the vessel wall, have been the subject of particular study [1, 2]. Their role is supported by shear-responsive behaviour of the endothelial cells (ECs) that line the arteries [3].

WSS is not the only mechanical factor to exhibit variation between arterial sites. Differences in intramural stress and strain also occur [4,5,6,7,8,9], and again can have a direct effect on cell function [10]. However, they have received much less attention, perhaps due to a lack of suitable techniques. They occur throughout the thickness of the wall, arise principally from blood pressure rather than flow, and vary cyclically as a result of the pulsing intraluminal pressure.

One argument for considering intramural stress in atherogenesis is the absence of lesions in the low-pressure venous system, despite its low WSS and similar blood lipid levels to arteries. When veins are used as arterial bypass grafts, and thus subjected to high pressure, they do develop atherosclerosis. Conversely, when arteries are transferred to the venous system they do not become diseased [11]. Sheathing of venous grafts limits disease [12]. Consistent with this, vertebral arteries exhibit a “rhythmic localisation” of atherosclerosis; lesions occur in the segments that are free to expand, but are absent where the artery passes through the bone canal [13, 14]. Similarly, the internal carotid artery is protected where it passes through the canal at the base of the skull [15]. Lesions in coronary arteries abruptly cease where these vessels enter the heart muscle, or where “myocardial bridges” occur [16]. During systole, the tunnelled segment undergoes compression due to contraction of the surrounding myocardium; external tissue pressures approach or even exceed intravascular blood pressure at this time [17]. Similarly, although constrictive cuffs placed around the carotid artery are purported to spare the cuffed segment through increased WSS [18, 19], we have shown that their effect may partly reflect altered strain [20].

Arterial branch ostia are analogous to openings in pressure vessels and thus subject to local stress concentrations. Stress/strain distributions around branch mouths will be complicated by the generally elliptical shape of the opening and the gently curving inflow tract upstream versus the relatively sharp flow divider downstream. When loaded to breaking, the descending thoracic aorta ruptures first at the origins of the intercostal arteries [21]. Thubrikar et al. [22] showed experimentally that lesion formation downstream of the renal artery branch in rabbits can be prevented by restricting vessel expansion. An age-dependent shift in the pattern of atherosclerosis occurs around the origins of branch arteries in the descending thoracic aorta, in rabbits as well as in people. Lesions occur downstream of branch ostia in immature vessels, moving to the lateral and then the upstream margins with age [23]. Following Thubrikar’s work, it is plausible that intramural stress and/or strain play a role in at least the development of the downstream lesions.

Capturing distributions of arterial strain is not straightforward. Finite element methods for numerically modelling stress and strain require detailed knowledge of the tissue architecture and mechanical properties of the individual tissue components, which are hard to obtain. *Ex vivo* mechanical testing disrupts the non-uniform tethering of the vessel. *In vivo* imaging of strain, for example by using ultrasound to detect changes in the pattern of scatterers, may ultimately provide a solution but currently has limited spatial resolution and is restricted to two dimensions; in animals, it requires anaesthesia, which is likely to lower mean and pulse pressure.

Here we describe a new technique that involves microscopical imaging of the impressions left by endothelial cell nuclei on plastic casts of the vascular lumen, made at different pressures. Because the casting method is destructive, different vessels need to be used at the different pressures and hence the patterns cannot be compared directly; new theoretical developments are presented to show how the distances between nuclei depend on pressure. The efficacy of the theory is demonstrated using simulated datasets and the method is then applied to the rabbit aorta, where it can be tested by comparison to the difference in diameter of the casts caused by changing the casting pressure. A novel approach is presented for obtaining nuclear locations from casts via confocal laser scanning microscopy (CLSM) rather than the conventionally-used scanning electron microscopy (e.g. [24]).

## Theoretical Background

**Fig. 1:**
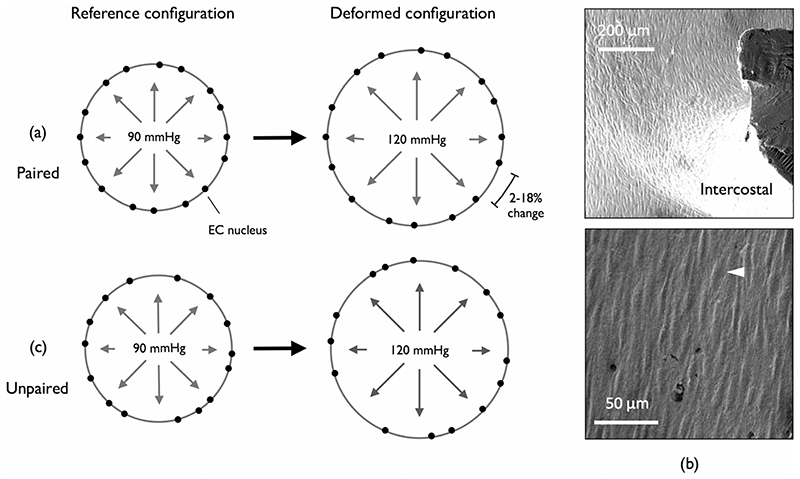
Strain from EC nuclear spacing - idealised vessel cross section. EC nuclei distributed around the circumference serve as strain markers. (**a**) When the reference and deformed configurations are paired, all inter-nuclear spacings increase with applied pressure; a 2–18% change is expected for large arteries. (**b**) SEM image of a corrosion cast of the rabbit aorta (example EC nuclear impression highlighted, white arrowhead). (**c**) When the reference and deformed configurations are unpaired, the average spacing increases with applied pressure

It is reasonable to assume that changes in the relative spacing between endothelial cell (EC) nuclei, arising due to cyclic changes in blood pressure, reflect changes in intimal strain (Fig. 1(a)). Vascular corrosion casts retain sub-cellular features of the endothelium; in particular, the protruding EC nuclei leave surface impressions (Fig. 1(b)). The destructive nature of the technique means that multiple casts cannot be obtained for the same vessel (the data are “unpaired”) but a statistical comparison of local inter-nuclear spacings between casts prepared at two different pressures should indicate local *in vivo* strains (Fig. 1(c)).

We wish to know how far apart each nucleus is from its surrounding neighbours and how this changes with increased pressure. The problem can be conceptualised as a nearest neighbour (NN) analysis, with the nuclei considered point-like events. Event-event distance methods of strain analysis are routinely used in geology to determine bulk strain in rocks, where the centres of individual grains serve as natural strain markers (first introduced in [25]). Similar to the nuclear spacing problem considered here, for a given deformed rock sample, there is no explicit information about the arrangement of grains before deformation. Derived strain estimates lack robustness though, suffering under anisotropy or requiring subjectivity and graphical methods [26–29]. For arteries, anisotropy is expected even in non-branching segments; there is negligible variation in vessel length over the pressure pulse but circumferential strain ranges from 2 to 18% [30]. Our analysis is an extension of the method of Lisle [31]. NN-based methods of strain analysis are not typically applied in a biological context so the theory will first be discussed in detail, highlighting that the analysis is not trivial.

Nuclear strain markers will be considered point-like events. Let *p* = {*p*_1_, …, *p*_*n*_} be a 2D point pattern observed in a square window *S* ⊆ *ℝ* ^2^, where *n* is the number of points. Though the following concepts can be extended to 3D, a 2D approximation of EC nuclear arrangement is reasonable if the size of the study region is small compared to the diameter of the vessel. We assume that *p* is obtained by intersecting a realization of a simple point process *P* with *S*, and that *S* is bounded with area [*S*]. *P* is stationary if its distribution is invariant under shifts in *ℝ* ^2^, and isotropic if its distribution is invariant under rotations around the origin. The intensity of *P* is given by *γ* = *n*/[*S*].

It is helpful to introduce the concepts of random and regular point processes and their simulation. The simplest spatial point process is complete spatial randomness (CSR, [32]). In this process, a point event has the same probability of occurring anywhere, and there is no interaction between events. Regularity in point patterns arises through negative interaction (“inhibition”) between events. This typically occurs due to the physical size of the objects that the point events represent. A simple 2D inhibition process is equivalent to the random packing of non-overlapping discs of diameter *δ*, where point events mark the disc centres. One simulation strategy for such a process is Sequential Spatial Inhibition (SSI, [33]). Candidate events are generated sequentially, rejecting those that are within *δ* of a previously accepted event. Both CSR and SSI processes are stationary and isotropic. We expect *P* to display regularity for nuclear point patterns as ECs are well packed, finite sized objects.

For simplicity, let us consider *P* subjected to strains aligned with the *x* and *y*-axes (no shearing). We assume strain is statistically homogenous over *S*. ***λ =*** (*λ*_*x*_, *λ*_*y*_) is the vector of applied stretch ratios; *λ* = *ℓ*/*L* where *ℓ* and *L* are the deformed and initial lengths respectively. Let *p* ′ = {*p*′_1_, …, *p*′_*n*′_} be a realization of the point process *P* subjected to ***λ*** observed in the square window *S*′ (the prime denotes the deformed configuration). When the deformed and reference windows have the same area ([*S*′] = [*S*]), *n*′ does not have to equal *n*. In the deformed configuration, the intensity of P is given by,

(1) $$\gamma^{\prime }=\frac{\gamma }{\lambda_{x}\lambda_{y}}$$

### Simple NN-Analysis

**Fig. 2:**
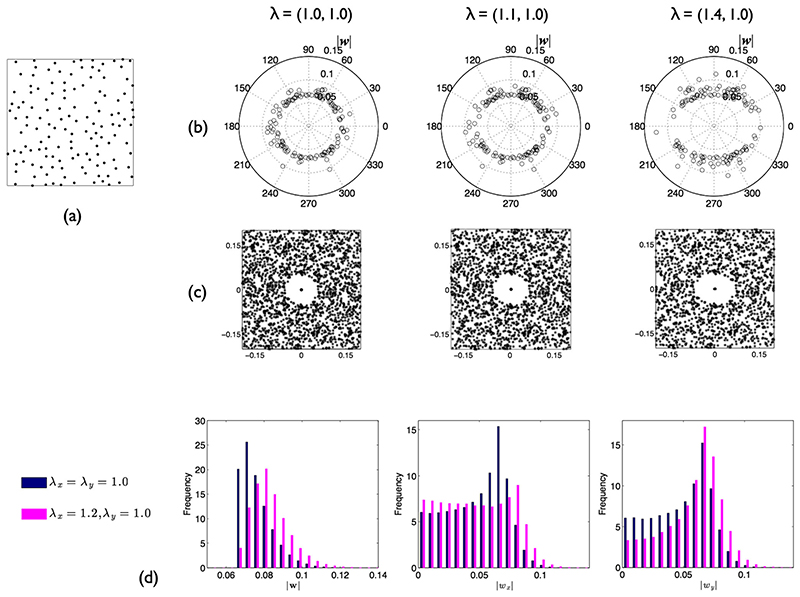
Ramsay and Fry methods. (**a**) Example SSI point pattern on a unit square (*n* =135, *δ* = 0.05). (**b**) Polar plots of NN orientation against distance for pattern in (**a**) subjected to anisotropic stretch. (**c**) Corresponding Fry plots. (**d**) Probability distributions of NN distance for SSI process (*n* =135, *δ* = 0.05, average of 200 realisations). Left: |***w***|. Middle: |*w*_*x*_|. Right: |*w*_*y*_|. Distributions are shown without (blue bars) and with (pink bars) anisotropic stretch applied

Strain in two dimensions can be represented by an ellipse. Under homogeneous finite strain, a circle is deformed into an ellipse with the length of its major and minor axes proportional to the principal stretches. Ramsay [25] reasoned that finite strain could be estimated by measuring the length and orientation of the vectors connecting each point with its NN $\left(w_{i}^{\prime }=(w_{i,x}^{\prime },w_{i,y}^{\prime }),i=\{1,\ldots ,n^{\prime }\}\right)$. Assuming that before deformation, points represented an isotropic inhibition process (their exact arrangement is unknown), distances in a given direction should scale in a manner directly proportional to the stretch in that direction. A polar plot of orientation against distance can provide a visual estimate of the ellipticity and orientation of the strain ellipse (Fig. 2(b)).

However, problems arise from the use of NNs to estimate distance, since NNs are not always strain invariant [28]. Under anisotropic strain, points are pulled apart, whilst being brought closer together in the perpendicular direction. Even if the arrangement of points before deformation is known, the NN of any point is not guaranteed to remain so after stretch. In extreme cases, Ramsay’s plot can reveal an ellipse with its long axis perpendicular to the principal stretch direction. More generally, compared with the reference configuration, ***w***′ orientation becomes biased towards the axis orthogonal to the direction of principal stretch.

Although simple NN-analysis returns erroneous estimates under anisotropy, stretch estimates can be calculated as follows.

So far, it has been assumed that *P* is isotropic and regular before deformation. (Without *a priori* knowledge of the point process, it is impossible to determine strains.) Due to the destructive nature of corrosion casting, nuclear point data cannot be obtained for the same vessel at two different pressures. However, provided that deviations in the spatial pattern of EC nuclei are sufficiently small between individual rabbits at a given location and pressure, ***λ*** can be estimated as the ratio between the mean NN distance in *S*′ and that in *S*. For *k* independent realisations of *P* in both *S* and *S*′, the estimates of stretch ratio in the *x* and *y*-directions are given by,

(2) $${\hat{\lambda }}_{x}=\frac{\sum_{j=1}^{k}|w_{x}^{\prime }{|}_{j}}{\sum_{j=1}^{k}|w_{x}{|}_{j}}\;{\hat{\lambda }}_{y}=\frac{\sum_{j=1}^{k}|w_{y}^{\prime }{|}_{j}}{\sum_{j=1}^{k}|w_{y}{|}_{j}}$$

where for one realisation of *P* in *S*, the mean orthogonal components are given by,

(3) $$|w_{x}|=\frac{\sum_{i=1}^{n}|w_{i,x}|}{n}\;|w_{y}|=\frac{\sum_{i=1}^{n}|w_{i,y}|}{n}$$

and similarly for *S*′. Because not all ***w***_***i***_ are independent observations, in practice a subset of size *m* is extracted by including ***w*** for mutually nearest neighbours only once. For increasing *k* in equation (2) one would expect the precision of $\hat{\lambda }$ to increase.

### Combined Retro-Deformation and NN-Analysis

Rather than restricting analysis to just NN vectors, the graphical method developed by Fry [26] utilises the interactions between all point events to determine the strain ellipse. A plot is created as follows. The entire set of points is translated so that one lies at the origin. This is repeated for another point and the translated dataset superimposed on that previously obtained. Continuing this process builds up the Fry plot (Fig. 2(c)).

For regularly arranged points, there is an obvious circular exclusion zone whose radius is governed by *δ*. If ***λ*** is anisotropic, this exclusion zone instead becomes a strain ellipse. Several automated approaches have been developed for fitting an ellipse to the central vacancy in the Fry plot [e.g. 27, 29] but real point patterns rarely generate a sharp central vacancy, leading to some subjectivity in the best choice of ellipse. In addition, a sufficiently large number of points is required to accurately capture the ellipse perimeter, limiting spatial resolution.

More recently, the concept of retro-deformation was put forward by Lisle [31]. *p*′ is subjected to a series of trial “de-strains” (***λ***_***r***_), equivalent to imposition of a strain ellipse with a given principal axis ratio (*λ*_*r*, 1_/*λ*_*r*, 2_) and orientation (φ, defined anticlockwise from the *x*-axis). Under the assumption that points before deformation represent the centres of well packed, similarly sized objects, the optimal de-strain is that for which the characteristics of the Fry plot satisfy isotropy and regularity conditions; the inverse of this retro-deformation defines the strain ellipse.

Rather than use all event-event distances and the Fry plot, here, for simplicity, we assess the optimal de-strain by a simple NN-based measure of isotropic regularity:

(4) $$\frac{\left|\text{w}\right|}{P(|\text{w}|,5)}$$

where |***w***| and *P*(|***w***|, 5) are, respectively, the mean and 5th percentile of the distribution of |***w***| over the window *S*. This measure arises from observations of the distribution of |***w***| for an SSI process (Fig. 2(d)); it is left truncated due to the imposed ***δ***. After anisotropic stretching, the distribution spreads rightwards, resulting in an increase in the ratio between the mean and minimum value. To limit the sensitivity to outliers, the minimum is replaced by the 5th percentile. The optimal de-strain is that which returns the minimum regularity index. Note that for purely isotropic scaling, the optimal de-strain is zero; NNs are already unbiased. For a CSR process, the expected value of the index is approximately 3.98, regardless of changes in intensity associated with retro-deformations. This may be estimated by recognising that for a CSR process, NN distances follow a Rayleigh distribution with scale parameter 1/2*πγ*.

As reported by Lisle, multiple de-strain solutions may exist, leading to ambiguity in the strain estimate. For extension in one direction accompanied by shortening perpendicular, both compression along the primary stretch axis or, conversely, extension along the secondary stretch axis would restore the pre-deformation arrangement (there are paired minima). We note that this issue can be simply addressed by applying the de-strained NN connections to the deformed state, to calculate a pseudo NN vector. ${\text{v}}_{\text{i}}^{\prime }$ connects the *i*th point to its pre-deformation NN; both points lie in *S*′. If it is assumed that *P* is isotropic and regular before deformation, ***λ*** for a single realisation of *P* subjected to finite strain may be estimated as the ratio between $\left|v_{x}^{\prime }\right|$ and |*w_x_*|, and similarly for *y*. Note |*w_x_*| represents the *x*-component of the NN vector in the optimal de-strained configuration. This approach overcomes the error encountered in the simple NN method due to bias in NN vector orientation when strains are anisotropic.

So far, it has been assumed that *P* is isotropic and regular before deformation. However in the case of EC nuclei, *P* exhibits anisotropy even in the absence of applied stretch, because ECs elongate and align with flow direction [3]. Therefore, retro-deformation must be applied in both the deformed and reference configurations to ensure no bias in NN vector orientation. Equation (2) can then be modified to yield,

(5) $${\hat{\lambda }}_{x}=\frac{\sum_{j=1}^{k}|v_{x}^{\prime }{|}_{j}}{\sum_{j=1}^{k}|v_{x}{|}_{j}}\;{\hat{\lambda }}_{y}=\frac{\sum_{j=1}^{k}|v_{y}^{\prime }{|}_{j}}{\sum_{j=1}^{k}|v_{y}{|}_{j}}$$

where $|v_{x}|=\sum |v_{i,x}|/m$ and $|v_{y}|=\sum |v_{i,y}|/m$ and similarly for ***v***′. *m* = *n* if mutual NN duplicates are not removed.

## Simulation Studies

The proposed stretch estimates were assessed for accuracy and precision using artificially generated datasets. All simulations were performed using Matlab 2017b (MathWorks, Inc).

### Study Region and Point Process Parameters

**Fig. 3:**
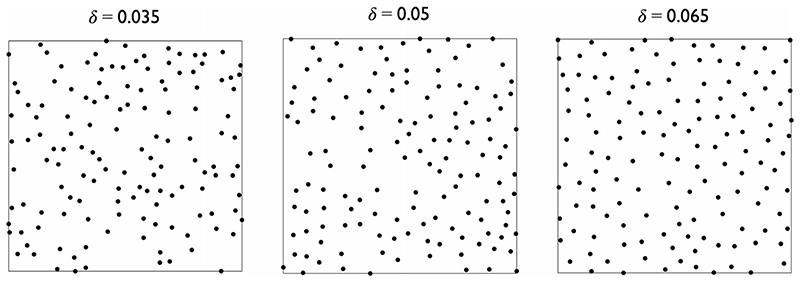
Realisations of SSI processes on a unit square with different *δ*, *n* =135

For simplicity the study region is defined as a 2D unit square, with *n* = 135 in the reference configuration (derived from data presented in [34, p. 251] where rabbit nuclear data were averaged over regions 250x250μm). Variance in *n* was not modelled in order to keep packing intensity (T = *γπδ* ^2^/4, [27]), and hence regularity, constant. It is assumed that all events are recorded i.e. there are no missing data. Isotropic regular point patterns were generated using SSI [28] with *δ* = 0 (CSR), 0.02, 0.035, 0.05 or 0.065 (examples of which are shown in Fig. 3); these can be related to the region-of-interest (ROI) in [34] by scaling by 250 μm. Edge effects are ignored. For inhibition point processes, the interaction between events sited near the boundary and those that would exist beyond it are thus neglected, but this ensures that *n* can be kept constant when required. The majority of simulations considered the case of ***λ =*** (1.1,1.0), which is within the expected physiological range [30].

### Optimal Retro-deformations

We first test the robustness of the regularity index (equation (4)) in returning the optimal retro-deformation. 300 SSI point patterns were generated and deformed. Trial retro-deformations were applied by imposing a strain ellipse with a given principal axis ratio (*λ*_*r*, 1_/*λ*_*r*, 2_) and orientation (φ, defined anticlockwise from the *x*-axis). *λ*_*r*, 1_ ranged from 0.5 to 2.0, increasing in increments of 0.025, whilst *λ*_*r*, 2_ was kept fixed at 1. φ ranged from 0 to 180°, increasing in increments of 2°. The trial which minimised the regularity index for each pattern was noted. Using the optimal retro-deformation, in each case ${\hat{\lambda }}_{x}$ and ${\hat{\lambda }}_{y}$ were computed according to equation (4) with *k* = 1. As the reference patterns were already isotropic, retro-deformation was only applied in the deformed configuration.

**Fig. 4:**
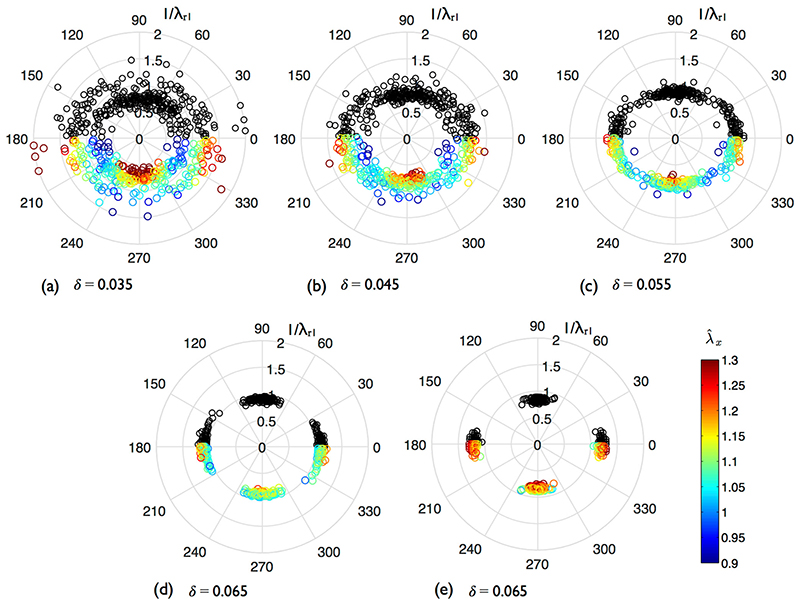
Optimal retro-deformations of anisotropically stretched realisations of SSI processes with different *δ* (*n* =135). 300 point patterns were generated for each stretch case. Black markers (0–180°): φ and 1/*λ*_*r*, 1_ which minimised the regularity index; (**a**–**d**) ***λ =*** (1.1,1.0). (**e**) ***λ =*** (1.2,1.0). 180–360°: the same results are shown but coloured by ${\hat{\lambda }}_{x}$, computed using equation (4) and rotated by 180°

Figure 4(a–d) displays the optimal retro-deformations when ***λ =*** (1.1,1.0) for increasing *δ*, plotted in black with polar coordinates (φ, 1/*λ*_*r*, 1_). Taking symmetry into account, points should cluster around 0 and 180° at a radius of 1.1 – or alternatively around 90° at a radius of 1/1.1 – if the regularity index is robust. (Whether φ is approximately equal to 0, 90 or 180° is determined by numerical precision). The lower half of each plot shows the optimal retro-deformations coloured by ${\hat{\lambda }}_{x}$; due to symmetry of the solution space, points are simply rotated by 180°.

When δ = 0.035, solutions do not cluster about specific values of *λ*_*r*, 1_ or φ, reaffirming that as *P* tends towards randomness, the optimal de-strain cannot be detected. With increasing δ, de-strain solutions gravitate towards their expected values. Their precision also increases with stretch (Fig. 4(e), δ = 0.065, ***λ =*** (1.2,1.0)). Despite the spread of optimal destrains when δ = 0.035, there is still sufficient interaction between the points that mean ${\hat{\lambda }}_{x}$ and ${\hat{\lambda }}_{y}$ (data not shown) yield reasonable values of 1.118 and 1.010 respectively. For stretches of *λ*_*x*_ = 1.1 and 1.2, the mean ${\hat{\lambda }}_{x}$ are 1.101 and 1.199, with standard deviations 0.041 and 0.052, respectively. Plots are not shown for ${\hat{\lambda }}_{y}$, but the means for both cases are 0.999 and 1.002.

### Anisotropic Stretch of SSI and CSR Processes

We first look at the case of paired data for *k* = 1. A single point pattern was subjected to *λ*_*x*_ = {0.1, …, 3} and *λ*_*y*_ = 1; stretch estimates were computed at each ***λ*** using the entire dataset ([*S*′] ≠ [*S*]). This was repeated for a further 299 patterns to establish means and 95% simulation envelopes. A simulation envelope is defined by ranking at each ***λ***, the values of $\hat{\lambda }$ for each repeat; the lower and upper envelopes are simply the 2.5th and 97.5th percentiles, respectively.

**Fig. 5:**
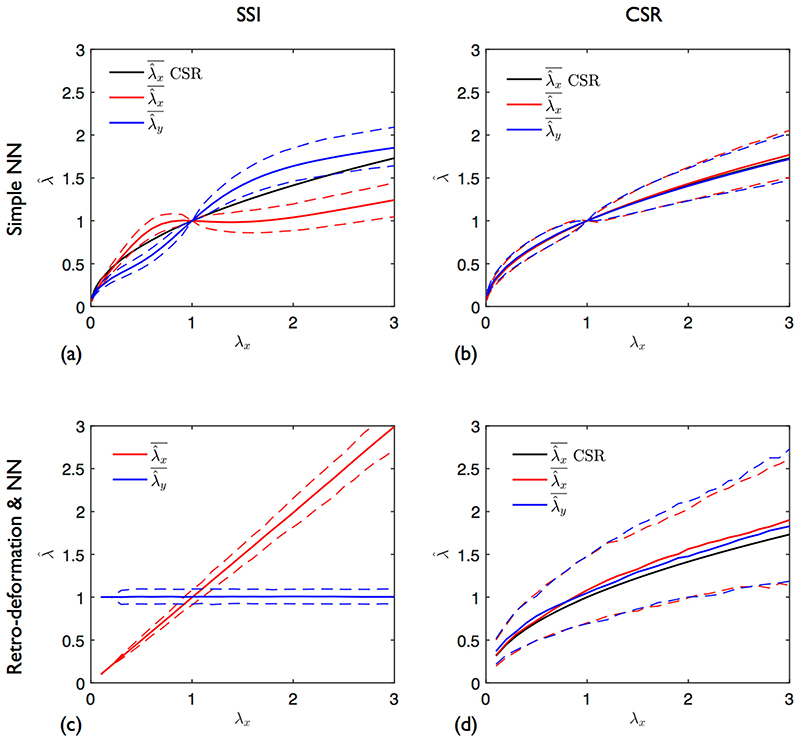
Variation in estimates with applied anisotropic stretch. *λ*_*x*_ = {0.1,…,3}, *λ*_*y*_ = 1. ${\hat{\lambda }}_{x}$ and ${\hat{\lambda }}_{y}$ (solid lines): means of 300 deformed point patterns. Dashed lines: 95% envelopes. (**a**), (**b**) Simple NN analysis. (**c**), (**d**) Retro-deformation plus NN analysis. (**a**), (**c**) SSI process: *n* = 135, *δ* = 0.065. (**b**), (**d**) CSR process: *n* = 135. ${\hat{\lambda }}_{x}$ *CSR* is the result expected under CSR ($\sqrt{\lambda_{x}}$)

Figure 5(a), (b) shows the results using simple NNs for δ = 0.065 and 0 (CSR), respectively. The poor performance of this earlier method is clearly evident; ${\hat{\lambda }}_{x}$ and ${\hat{\lambda }}_{y}$ do not follow the applied ***λ***. For (Fig. 5(a)), the flattened portion of the curve between ${\hat{\lambda }}_{x}$ = 0.8 and 1.5 is due to the change in nearest neighbour connections. Beyond this region, further connection changes are minimal so the estimate starts to increase (or decrease for *λ*_*x*_ < 1). Considering the denominator in equation (1), it is clear that ***λ*** = (*λ*_*x*_, 1.0) gives the same change in *γ* as $\lambda =(\sqrt{\lambda_{x}},\sqrt{\lambda_{x}})$. For a purely CSR process, both ${\hat{\lambda }}_{x}$ and ${\hat{\lambda }}_{y}$ follow the theoretical curve $\sqrt{\lambda_{x}}$. Edge effects start to become noticeable at the stretch extremes. ${\hat{\lambda }}_{x}$ increases at a slightly higher rate than ${\hat{\lambda }}_{y}$ for *λ*_*x*_ > 1; the opposite occurs for *λ*_*x*_ < 1.

In contrast, when using our new combined retro-deformation and NNs approach, ${\hat{\lambda }}_{x}$ and ${\hat{\lambda }}_{y}$ do follow the applied ***λ*** for δ = 0.065 (Fig. 5(c)). When δ = 0, the stretch estimates again follow $\sqrt{\lambda_{x}}$, demonstrating that neither method returns accurate estimates if the underlying point process is random.

### Anisotropic Stretch of SSI Process: How Many Replicates are Required?

We now consider the case of unpaired data with *k* ≥ 1. We want to determine how large *k* needs to be for ${\hat{\lambda }}_{x}$ to become significantly different from zero. A simulation was performed as follows. At *k* = 1, two independent realisations of *P* were generated, one of which was then deformed by ***λ***. Combined retro-deformation and NN analysis was then applied to both and stretch estimates computed according to equation (5). Area was kept fixed regardless of strain ([*S*′] = [*S*]), so that intensity decreased with stretch. This was repeated up to *k* = 50; at the end of each iteration *k*, the cumulative moving average of $\hat{\lambda }$ was computed. The process was repeated 200 times to calculate means and 95% simulation envelopes.

To separate the effect of variance between patterns from errors associated with retro-deformation, a second simulation was performed. In this, retro-deformation was not applied to point patterns in either configuration. Instead, for each deformed pattern, neighbour connections were taken from the NNs of the same pattern before stretch was applied i.e. pre-deformation NNs were treated as known. Note reference patterns are already isotropically regular.

**Fig. 6:**
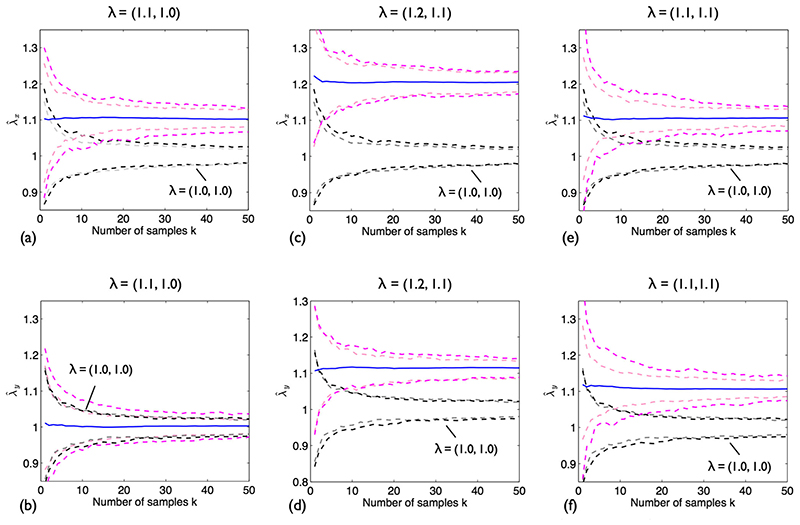
Stretch of an SSI process; combined retro-deformation and NN analysis. *n* = 135, *δ* = 0.05. Top row: ${\hat{\lambda }}_{x}$. Bottom row: ${\hat{\lambda }}_{y}$. (**a**), (**b**) ***λ =*** (1.1,1.0). (**c**), (**d**) ***λ =*** (1.2,1.1). (**e**), (**f**) ***λ =*** (1.1,1.1). Solid blue lines: means of 300 simulations. Pink dashed lines: 95% envelopes. Black dashed lines: 95% envelopes for ***λ =*** (0, 0). 95% envelopes obtained for known NNs shown as paler dashed lines

Figure 6 show the 95% simulation envelopes and ${\hat{\lambda }}_{x}$ and ${\hat{\lambda }}_{y}$ means for δ = 0.05 and three different ***λ***. The envelope for each estimate when ***λ*** = (1.0,1.0) is also plotted. The envelopes resulting from the second simulation study – no retro-deformation but known pre-deformation NNs – are shown alongside as paler dashed lines.

**Fig. 7:**
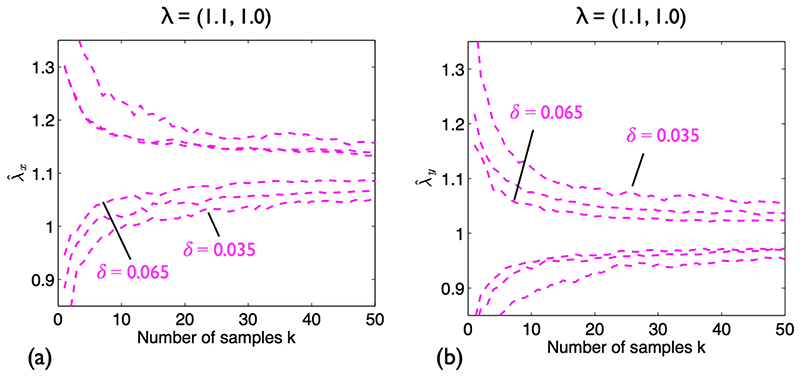
Anisotropic stretch of SSI processes with varying *δ*, *n* = 135; combined retro-deformation and NN analysis. Pink dashed lines: 95% envelopes. ***λ =*** (1.1,1.0). (**a**) ${\hat{\lambda }}_{x}$. (**b**) ${\hat{\lambda }}_{y}$

It is clear that as *k* increases, the precision of the estimates increases. When ***λ*** = (1.1,1.0) (Fig. 6(a), (b)), in the *x*-direction, envelopes for the stretch and no stretch cases no longer overlap at *k* = 15; the result for ${\hat{\lambda }}_{y}$ is not significant, as expected. When ***λ*** = (1.2,1.1) (Fig. 6(c), (d)), ${\hat{\lambda }}_{x}$ and ${\hat{\lambda }}_{y}$ are slightly overestimated: 1.21 and 1.12, respectively. Further simulations are required to establish if this is a consistent finding or just due to an insufficient number of repeats and/or inadequate sample size. Although the stretch and no stretch envelopes rapidly deviate for the *x*-direction (*k* = 4), double the sample size is required to separate these envelopes in the *y*-direction. Lastly, it is confirmed that retro-deformation does not adversely affect the result for isotropic stretch (Fig. 6(e), (f)); note that simple NN analysis will return the correct value in this case. In general, combined retro-deformation and NN analysis leads to slightly wider envelopes than the case where NNs are known. Figure 7 shows the effect of varying δ for ***λ*** = (1.1,1.0). Decreasing δ broadens the envelopes, implying larger *k* are required to attain significance.

## Strain in the Descending Thoracic Aorta of Immature Rabbits

### Methods

#### Animals

All animal procedures complied with the Animals (Scientific Procedures) Act 1986 and were approved by the local ethical review panel of Imperial College London. Six immature male New Zealand White rabbits (HSDIF strain; Harlan, UK) were used; they were divided into two equal groups (diastolic, systolic). For the diastolic group, ages were 9, 9 ½ and 11 weeks with weights 1.68, 1.84 and 1.83 kg, respectively. For the systolic group, ages were 9 ½, 9 and 9 weeks with weights 1.69, 1.72 and 1.96 kg, respectively.

#### Vascular corrosion casting

Each animal received heparin (2000 USP units, *iv*) and was subsequently euthanised by an overdose of sodium pentobarbitone (Euthatal, Rhone Merieux; 0.8 ml/kg, *iv*). After placing the animal in the supine position, the thorax was opened by a midline incision and the rib cage retracted. The aorta was cannulated at the level of the diaphragm for the inflow and at the top of the aortic arch, after the 3 main branches, for the outflow, using approximately 20 cm lengths of 3 mm-diameter PVC tubing connected to closed three-way taps, all filled with saline. Once tied in place, each cannula was further secured with cyanoacrylate glue to prevent leaks.

Methyl methacrylate resin (Batson’s #17, Polysciences, Inc., Germany) was prepared according to the manufacturer’s instructions and drawn into a syringe. Bubbles were allowed to rise to the syringe tip for 5 min and were flushed out before perfusion. Meanwhile, a saline filled syringe attached to a pump (Pump 33, Harvard Apparatus, UK) and a digital manometer (HD570, Extech Instruments, USA) were connected to the inflow tap. The aorta was then flushed with the outflow tap open. The saline syringe was swapped for the one containing the resin, and the aorta perfused until resin appeared at the outflow. At this point, the outflow tap was closed and perfusion continued till the desired pressure was reached. For the diastolic group, this was 90 mmHg and for the systolic group, 120 mmHg. Pressure was maintained by manually stopping and starting the pump as required until the resin hardened i.e. the syringe pump stalled. On average, there was a 2 h gap between death and the start of resin perfusion.

After curing overnight, the casted aorta was dissected from the carcass and submerged in KOH (30% *w*/*v*) for 24 h to digest the remaining tissue. The cast was further cleaned with detergent (Decon90, Decon Laboratories Ltd., UK) prior to use.

#### Confocal laser scanning microscopy (CLSM)

On each cast, the intercostal arteries were trimmed back to short stumps so as not to obscure the aorta during imaging. Between successive pairs of intercostals, the dorsal surface was coated with a thin layer of highlighter ink (taken from a yellow Stabilo Luminator pen) down to the 4th pair. Capillary action caused ink to pool in the nuclear imprints, locally elevating signal intensity such that the indentations were easily differentiated from the surrounding surface, even with low power, low NA objectives.

The highlighted surface was imaged using an inverted Leica SP5 CLSM with motorised stage and 10x, NA 0.4 dry objective (488 nm excitation, 504-558 nm emission). Casts were attached to an adjustable slide holder at either end, mounted so that the circumferential and axial directions aligned with the *x* and *y* imaging axes, respectively. No coverslip was employed. Care was taken to ensure the cast surface was not axially tilted with respect to the imaging plane. 3D image volumes were obtained with a voxel size of 1.17 × 1.17 × 1.01 μm (*x*, *y*, *z*); a zoom of 1.3 was used to minimise spatial biases in intensity. The image *z*-axis was flipped so that the images represented the endothelium viewed *en face*, rather than an exterior view of the cast surface.

#### Image processing

All image data were processed using ImageJ (imagej.nih.gov/ij). Considering the ratio between the aortic diameter and ROI size, errors associated with surface curvature were ignored. The maximum intensity projection of the 3D image volume was taken to produce a 2D image; a 600x600μm ROI was then selected. To correct for the uneven intensity of the highlighted surface, a rolling ball filter (radius 5 pixels) was applied to generate a background image which was then subtracted from the original. The resulting image was then thresholded and features too small to be nuclei removed by application of the “Despeckle” filter; occasional apparently-touching nuclei were separated by manual editing. The centroids of the resulting features were computed; these points represented the nuclear centres. In total, 12 ROIs were selected from each pressure group, ensuring a minimum of 3 came from each animal.

#### Strain analysis

To perform the combined retro-deformation and NN strain analysis, point data were imported into Matlab 2017b (MathWorks, Inc). To prevent edge effects, a guard zone was constructed by selecting a 500 × 500 μm ROI within each point set. Only points inside this region were included in the analysis (total *n*) but their NN could lie in the guard zone.

Every point pattern (both systolic and diastolic) was subjected to over 50,000 trial retro-deformations to establish where in solution space the regularity index (equation (4)) was minimised. *λ*_*r*, 1_ ranged from 0.25 to 4.0 increasing in increments of 0.025 (*λ*_*r*, 2_ was fixed at 1), whilst φ ranged from 0 to 180° increasing in increments of 0.5° (angle defined anti-clockwise from the *x*-axis). The optimal retro-deformation was applied to the point pattern and unbiased NN connections determined; mutual NNs were included only once. Using the original point coordinates, the mean components of the vector ***v*** (***v***′ for the systolic group) were then computed for each pattern. Treating all datasets as independent (*k* =12), ${\hat{\lambda }}_{x}$ and ${\hat{\lambda }}_{y}$ were computed using equation (5) where the prime notation is used to signify the systolic group, *x* is the circumferential direction and *y*, the axial. For comparison, conventional NN analysis (no retro-deformation) was also performed, using equation (2). Note stretch estimates computed by either method represent cyclic stretch (i.e, the difference between systolic and diastolic pressure) rather than absolute stretch. To account for the use of multiple datasets from each rabbit, the significance of differences between the two pressure groups was assessed using nested ANOVA. The dataset locations were treated as random effects and *p* < 0.05 was taken as the criterion of significance.

*In vivo*, strain gradients would be expected near geometric discontinuities such as branches. To establish the efficacy of the proposed methodology in such a case, a single point pattern from the diastolic rabbit group (Fig. 9(a)) was subjected to a deformation gradient in the *x*-direction of the form *x*’ = *ax* ^2-^ + *x* (*y*’ = *y*), where *a* = 0.05,0.1. The ROI was scaled with stretch ([*S'*]≠[*S*]) to remove the effects of variable sample size. The ROI was divided into 4, 9 or 16 square boxes in the reference configuration; as with the ROI itself, the boundaries of these boxes deformed with the applied stretch. Stretch estimates were computed using the combined retro-deformation plus NN method. The optimal retro-deformations were refined by trialling the range *λ*_*r,1*_ ± 0.05 in increments of 0.005, and the range φ ± 3° in increments of 0.1°. This was found to be essential given the small sample sizes.

#### Measurement of aortic cast geometry

Vascular corrosion casts made at 90 and 120 mmHg were imaged with 27-μm isotropic resolution using a Skyscan 1172 desktop microcomputed tomography scanner. An isosurface representing the blood-wall interface was extracted and smoothed, and cylindrical coordinates were computed for the aorta, with software from the Vascular Modelling Toolkit (www.vmtk.org). The cylindrical mapping was used to calculate the axial spacing between intercostal artery branch mouths on the anatomical left and right sides, and aortic radii were computed as the mean maximum inscribed sphere radius between successive pairs of branches. There were five intercostal pairs on each cast so four values of aortic radius and of axial spacing on each side were obtained. It was not possible to obtain microCT data for one cast (rabbit C, 120 mmHg) so measurements were obtained using callipers (accuracy 0.01 mm).

### Results

Systolic casts were noticeably bigger than diastolic ones. Individual values for aortic radius and axial spacing between intercostal ostia are given in Supplementary Material Table s3. The mean aortic radius in the systolic group was 2.65 mm and 1.80 mm in the diastolic group (*p* = 0.73 × 10^−3^). Circumferential stretch was therefore 1.47. There was no significant axial stretch: axial stretch was 1.01 and 1.03 on the left and right side of the dorsal surface, respectively (*p* = 0.868 and *p* = 0.722.)

**Fig. 8:**
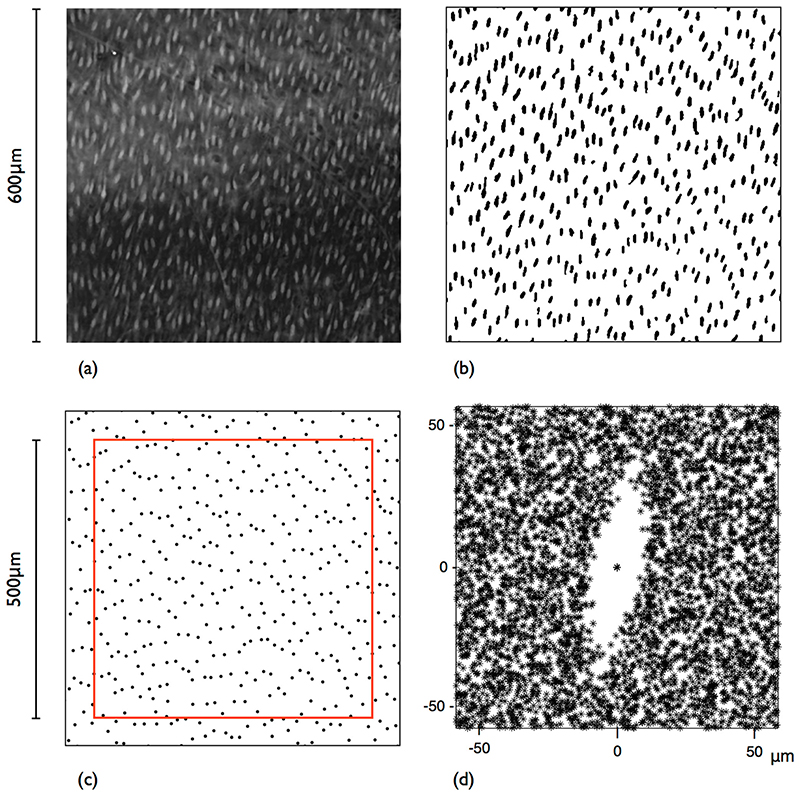
Segmentation of highlighted nuclear imprints. (**a**) A 600x600μm ROI from the maximum projection image of a CLSM scan. Blood flow direction is top to bottom. (**b**) After background subtraction, thresholding and despeckle filtering. (**c**) Point pattern obtained by computing the centroids of the segmented nuclear features shown in (**b**). Red box is the ROI used for strain analysis; exterior points lie in the “guard zone”. (**d**) Fry plot of the data in (**c**)

**Fig. 9:**
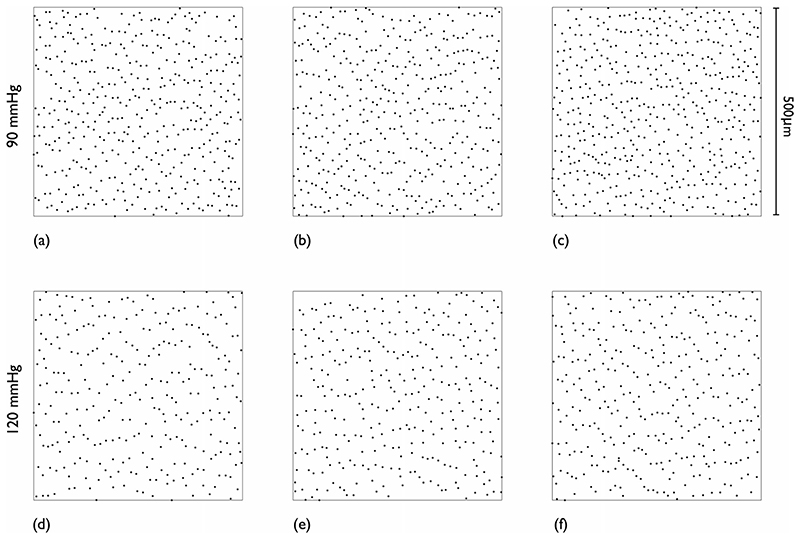
Example EC nuclear point patterns at each pressure. One pattern from each animal. Guard zone excluded. (**a**–**c**) 90 mmHg. (**d**–**f**) 120 mmHg. Blood flow direction is top to bottom

An example 600x600μm ROI maximum projection image is shown in Fig. 8(a); nuclear impressions are readily identified as bright elliptical objects against a darker background. The segmentation results are shown in Fig. 8(b). The centroids of the segmented nuclei are plotted in Fig. 8(c) with the smaller 500x500μm ROI overlaid (the area exterior to this is the guard zone). Further examples of the nuclear point patterns obtained (guard zone excluded, one from each animal) are shown in Fig. 9. Qualitatively, points appear closer together circumferentially than axially. There is a visible decrease in point intensity from the low to the high pressure group.

As the retro-deformation and NN strain analysis method assumes that points are not randomly arranged, a Fry plot (Fig. 8(d)) was generated for the point pattern shown in Fig. 8(c). There is an obvious exclusion zone, characteristic of regular point processes. Its elliptical shape is not strictly speaking the strain ellipse; it is the combined effect of strain and the anisotropic arrangement of the points due to flow-mediated EC elongation. The orientation of the elliptical exclusion zone compares with the orientation of the nuclei in the original image (Fig. 8(a)). From ellipses fitted to each of the segmented nuclear features, the average orientation was found to be 80.8°, defined anti-clockwise from the *x*-axis; the orientation of the exclusion zone is approximately 80°. Note that each point pattern could also be assessed for its underlying regularity through its departure from a CSR process (Diggle’s G function deviation test [32]).

**Fig. 10:**
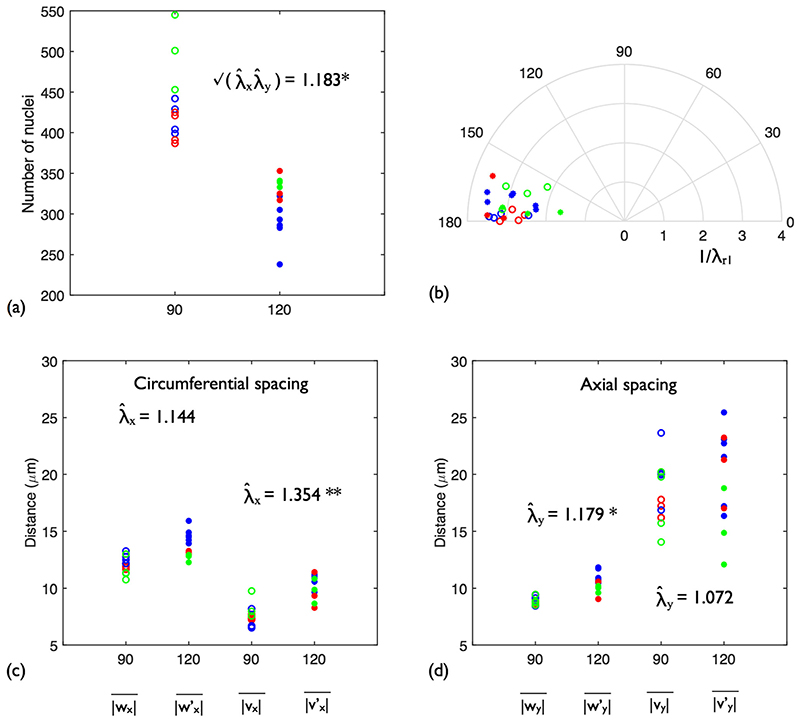
Strain analysis results for the rabbit descending thoracic aorta. Open circles: 90 mmHg. Filled circles: 120 mmHg. The data points are coloured blue, red or green according to which rabbit (A, B or C) the sample belonged to at each pressure. (**a**) EC nuclear density at each pressure. (**b**) Optimal retro-deformations for each point pattern. (**c**) Circumferential (*x*) spacing. |*w_x_*| and $\left|w_{x}^{\prime }\right|$ were computed using simple NN analysis. |*v_x_*| and $\left|v_{x}^{\prime }\right|$ were computed using combined retro-deformation and NN analysis. (**d**) Axial (*y*) spacing. *p < 0.05, **p < 0.001

Figure 10(a) shows the number of nuclei in each sample. The data points at each pressure are coloured blue, red or green according to the rabbit from which they derived. The mean of the systolic group was substantially higher than that of the diastolic group (435.5 vs 311.3; *p* = 0.0183).

Figure 10(b) shows the solution space of the optimal retro-deformation for each sample (colours as previously defined). Combining groups, the mean de-strain solution was *λ*_*r*, 1_ = 2.90, φ = 172° (the difference between groups was not significant). Considering the shape and orientation of the exclusion zone in the Fry plot shown above, the results are not surprising; the retro-deformations return the elliptical central vacancy to a circular one. The ratio between *λ*_*r*, 1_ and *λ*_*r*, 2_ could actually be thought of as a surrogate index of cellular shape. In addition, the retro-deformation solutions suggest that in the majority of samples, nuclei were aligned approximately 10° to the axial direction (confirmed by visual inspection of the images). This might be due to a consistent bias in mounting the casts on the confocal microscope, but results presented in [34, 35] indicate EC nuclei can be oriented in this manner down the dorsal side of the aorta, putatively due to vortical flow structures.

**Fig. 11:**
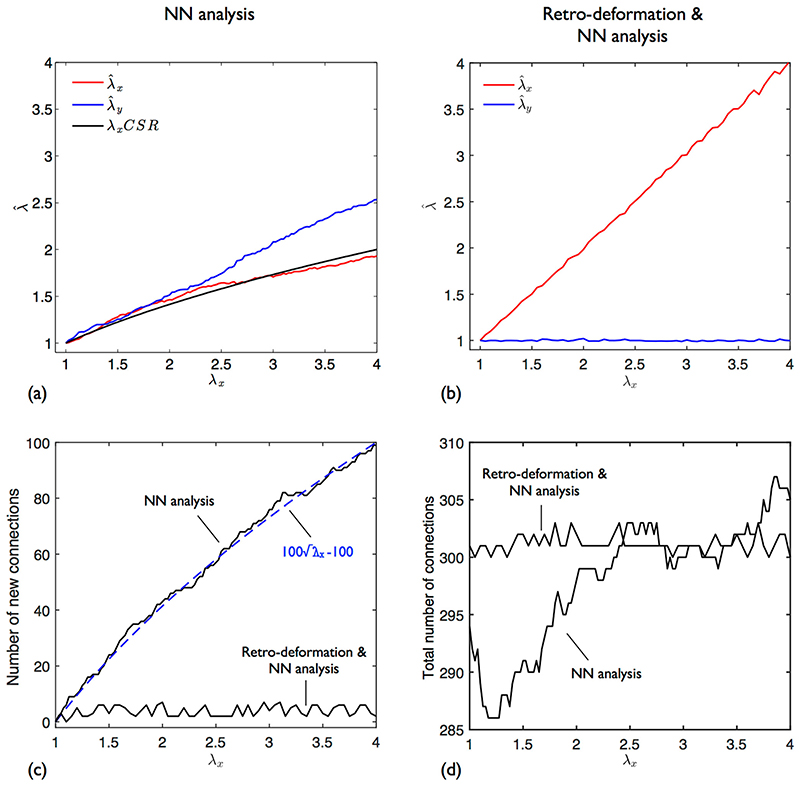
Anisotropic stretch of a single EC nuclear point pattern. *λ*_*x*_ = {1,…,4}, *λ*_*y*_=0. (**a**) Stretch estimates computed with simple NN analysis. Also plotted is the theoretical result for a CSR process. (**b**) Stretch estimates computed with combined retro-deformation and NN analysis. (**c**) Number of new neighbour connections identified (i.e. those connections that were not present at zero stretch) when using both methods. For simple NN analysis, the trend is proportional to $\sqrt{\lambda_{x}}$. (**d**) Total number of neighbour connections identified when using both strain analysis methods

Before presenting the nuclear spacing results, it is useful to consider the artificial stretch of one EC pattern. As shown previously for SSI processes in Section 3.2, for the conventional NN analysis, ${\hat{\lambda }}_{x}$ is less than expected whilst ${\hat{\lambda }}_{y}$ is greater (Fig. 11(a)). In contrast, using retro-deformation, ${\hat{\lambda }}_{x}$ follows the applied *λ*_*x*_ whilst ${\hat{\lambda }}_{y}$ stays at 1 (Fig. 11(b)); the minor deviations are due to the limited resolution of the trial de-strain solution space.

Curves for ${\hat{\lambda }}_{x}$ and ${\hat{\lambda }}_{x}$ obtained using simple NN analysis closely follow the solution expected for a CSR process of the same intensity $\sqrt{\lambda_{x}}$, up to *λ*_*x*_ ≈ 2.5. NN distances (|***w***|) for a CSR process follow a Rayleigh distribution; adding an inhibition distance simply truncates this (Fig. 2(d)). Due to this underlying similarity, it is perhaps not surprising that the estimates and the ideal CSR solution broadly follow the same trend. Gross deviations start to occur when nearest neighbour orientation switches from being predominantly circumferential to isotropic. As the pattern is deformed, NN connections change. The number of new connections formed (i.e. those connections that were not present at zero stretch) is plotted in Fig. 11(c) and is proportional to $\sqrt{\lambda_{x}}$. There is also a small increase in the total number of connections (Fig. 11(d)). Also shown are the equivalent results for the combined retro-deformation and NN analysis; there is minimal change in neighbour connection and total number of connections.

Figure 10(c), (d) shows the mean nuclear spacings computed for the diastolic and systolic groups using both strain analysis methods, and their associated estimated stretch ratios. In the circumferential direction, the simple NN analysis yielded |*w_x_*| and $\left|w_{y}^{\prime }\right|$ that were generally higher than the combined retro-deformation plus NN analysis counterparts (|*v_x_*| and $\left|v_{y}^{\prime }\right|$). The opposite was true in the axial direction. Both trends are due to the bias in NN orientation discussed above. Retro-deformation removes this bias, therefore reducing the *x*-components of the NN vectors whilst increasing the *y*-components. Comparison of the spacing metrics between groups showed that only axial spacing differences were significant for simple NNs (|*w_y_*| vs $\left|w_{y}^{\prime }\right|$, *p* = 0.0181), whilst only circumferential spacing was significant with combined retro-deformation and NN analysis (|*v_y_*| vs $\left|v_{y}^{\prime }\right|$, *p* = 8.71 × 10^−3^).

For simple NN analysis, the estimated stretch ratios ${\hat{\lambda }}_{x}$ and ${\hat{\lambda }}_{y}$ were approximately equal: 1.144 and 1.179 respectively. In contrast, the combined retro-deformation and NN method showed there was a greater circumferential stretch than axial: ${\hat{\lambda }}_{x}$ = 1.354 and ${\hat{\lambda }}_{y}$ = 1.072. When compared with the ratios between the mean cast dimensions from each group (1.47 circumferentially and 1.02 axially), the combined method appears to yield robust stretch estimates. Note that samples were taken from the dorsal side which, due to tethering to the spine, likely deforms less than the ventral side. Considering equation (1), stretch estimates can be related to the change in nuclear intensity by $\sqrt{\lambda_{x}}$ *λ_y_*. It is interesting to note that both methods return values similar to that obtained directly from the number of points; 1.162 for simple NN analysis and 1.205 for retro-deformation, compared with 1.183 from point number. Detailed results of the strain analysis using both methods are summarised in Tables s1 and s2 in the Supplementary Material.

**Fig. 12:**
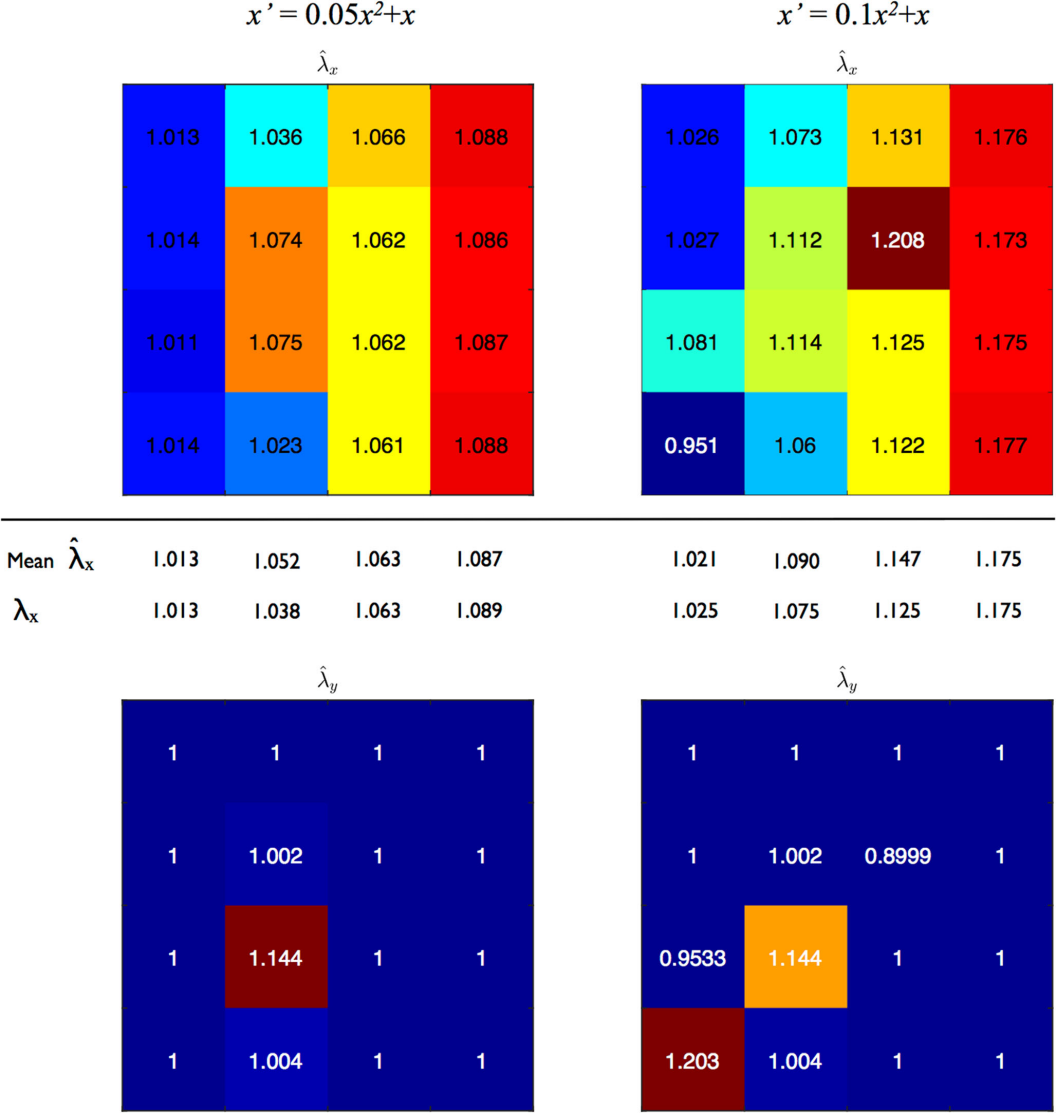
EC nuclear point pattern subjected to two linear deformation gradients. Each small box represents a ROI that was 125 × 125 μm in the reference configuration. Values within each box are stretch estimates computed with combined retro-deformation and NN analysis. Top row: ${\hat{\lambda }}_{x}$, column averages and expected values given below. Bottom row: ${\hat{\lambda }}_{y}$, expected value 1

Figure 12 shows maps of estimated stretch ratios when the point pattern in Fig. 9(a) is subjected to linear deformation gradients in the *x*-direction and the pattern divided into 16 square boxes for analysis. Despite the small sample sizes (each box is 125x125μm, with 22–30 nuclei per box), ${\hat{\lambda }}_{x}$ and ${\hat{\lambda }}_{y}$ are generally in good agreement with their expected values. (Seven out of sixteen column averages agree with the expected result to 3 decimal places.) Discrepancies occur in some places due to NN connection changes; with such a small sample size, just one change can have a large impact on the estimates. Even when using known NN connections to calculate stretch, maps display slight deviations due to point location and density (i.e. inadequate sampling; data not shown).

Increasing box size, whilst obviously limiting resolution of the deformation gradient, will not necessarily improve the result because the retro-deformation approach assumes stretch is homogeneous across the box. However, in this example, the gradient is still discernable when using 9 or even 4 boxes rather than 16 (Figures s4 and s5 in the Supplementary Material).

### Discussion

This study successfully demonstrated that pressure-induced stretch of the rabbit aorta can be determined by considering the relative change in nuclear spacing on corrosion casts made at two different pressures. Analysing nuclear point patterns obtained at diastolic and systolic pressures revealed that cyclic circumferential stretch is 1.35 on the dorsal surface of the thoracic aorta. This estimate was comparable to, but slightly less than, the change in mean cast (and thus vessel) diameter. The difference was likely due to perivascular tethering to the spine, restricting vessel expansion on the dorsal surface compared with the ventral. In contrast, axial stretch did not occur; in addition to axial prestretch [10], anchoring of the aorta to the spine also limits length distension.

The circumferential stretch was higher than anticipated [30]. The difference could result from an alteration of vascular tone, the lack of a time-varying pressure, or a systematic error in the measurement of pressure during casting. A *post mortem* change in vascular responsiveness is considered the most likely. Although aortic tone is still sensitive to pharmacological agents, at least in rats, for 2 h after death [36], alterations do occur: our preliminary work in mature rabbits showed that reducing the time between death and resin perfusion produces systolic casts that appear overly constricted, and significantly smaller in diameter than the immature casts presented here (data not shown). Further investigation is required to establish what casting conditions and time frames are required to capture the active mechanical response.

Arteries are constantly subject to a basal stretch, which is related to mean blood pressure, and to a cyclic stretch due to the pulse pressure. One limitation of our approach is that absolute stretch (i.e. relative to the stress-free configuration) cannot be determined. However, several studies have suggested that it is the pulse pressure, transduced by cyclic deformation of vascular cells, rather than the mean pressure that plays a key role in arterial properties such as remodelling [37, 38]. Physiological cyclic stretch levels increase EC proliferation [39, 40] and suppress apoptosis [41, 42], whilst supraphysiological levels stimulate apoptosis [41] and may lead to uncontrolled proliferation [40]. Cyclic stretch alters tight junction structure [43], so permeability is also likely affected. Uptake of nanoparticles is enhanced by acute cyclic stretch [44]. In addition, a reduction in cyclic wall motion inhibits hypertension-mediated atherosclerosis [45]. Hence measuring only cyclic stretch appears still to be useful for many physiological and pathological processes.

The geometric fidelity of the corrosion casts has been debated; the resin can shrink during the curing process in a nonlinear manner. Volume shrinkage as high as 13 to 20% has been reported for Batsons #17 [46], whilst others have reported negligible change [24, 47, 48]. Moore et al. [49] compared the accuracy of corrosion casts relative to *in vivo* MRI and found aortic radii agree to within 9%. Krakty et al. [50] concluded that if care was taken to maintain physiological pressures during resin infusion, reliable results could be produced. The manner in which the aorta was cast in the present study did maintain pressure; resin did not leak, or rapidly stopped leaking, from the vessels cut along the sternum, presumably due to their relatively small diameter and the high resin viscosity.

**Fig. 13:**
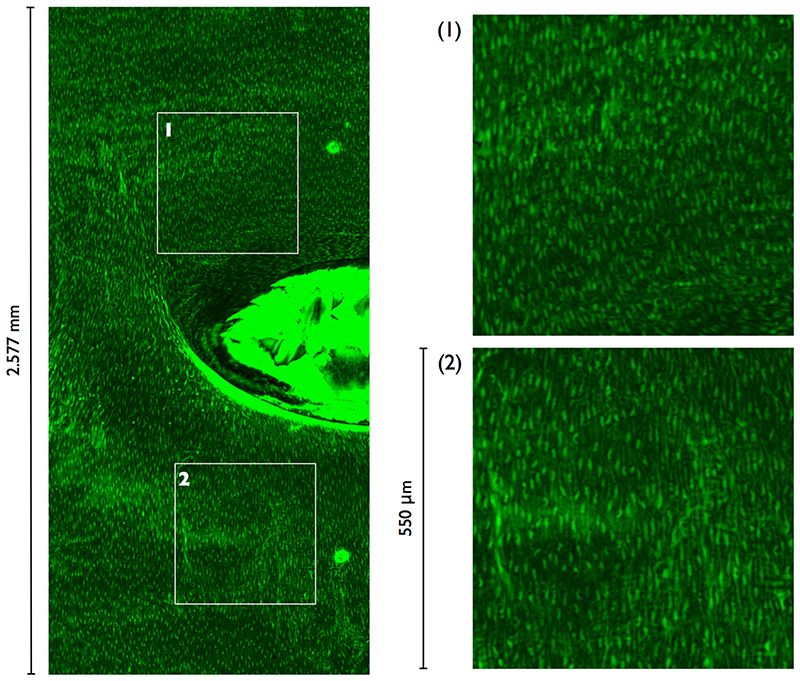
Maximum intensity projection from CLSM scan of an intercostal branch ostium using a 4x objective. Half the field-of-view is displayed. Magnified views of the marked 550x550μm ROIs are shown on the right. Blood flow direction is from top to bottom

Application of the new analysis technique to branch regions and consequent comparison of patterns of strain and disease may support or refute the hypothesis that the development of experimental atherosclerosis at these sites is influenced by mural strain. Extension to branch sites requires imaging much larger ROIs. Preliminary work has demonstrated that this is possible if a 4x objective is used (Fig. 13). Due to curvature effects, the location of nuclear centres could no longer be extracted from 2D maximum intensity projections.

Strain analysis at branch sites was not possible with the present casts as some areas around ostia did not contain endothelial impressions. The same applied for the ventral surface. (Higher circumferential stretch is expected here than on the dorsal surface.) This may be due to depressurisation of the aorta causing endothelial denudation. Pre-fixation has been suggested by some authors for improved endothelial imprints, prevention of extravasation of the resin and maintenance of tissue integrity [50–52]. Although aldehyde-based fixatives cause substantial tissue contraction, fixation with HgCl_2_ can accurately maintain *in vivo* dimensions, even of elastic vessels [53].

Extension to branch sites also requires determining the minimum resolution at which stretch can be resolved; around branch openings, strain and cellular orientation gradients ([4, 35]) are expected to be steep and ROIs should be sufficiently small that strain can be considered locally uniform. The metric for determining the optimal retro-deformation is based on a distributional assumption and may suffer under very small numbers of points, when the value of the 5th percentile is interpolated. Although the strain gradient test results were promising, further work may be required to establish a metric more suited to small ROIs. Furthermore, with smaller ROIs, more samples will be required to achieve the same level of precision. We have re-run the combined retro-deformation and NN analysis with a reduced ROI of 250x250μm; Stretch estimates became ${\hat{\lambda }}_{x}$ = 1.35 (*p* = 5.98 × 10^−3^) and ${\hat{\lambda }}_{y}$ = 1.17 (*p* = 0.19), in good agreement with the previous results for the larger ROI.

## Conclusion

Simplification of retro-deformation methods developed for geological studies, in novel combination with nearest-neighbour analysis from the same literature, has permitted the development of analytical methods that permit local arterial strain to be determined from the impressions left by endothelial cell nuclei on the surface of vascular corrosion casts made at different pressures, even though only one pressure can be examined in each vessel. Simulations showed that the method was accurate, precise and robust when applied to model data, unlike existing methods based on nearest neighbour distances alone; the latter give errors because the identity of the nearest neighbour is changed by the deformation itself. The method does not require unfeasibly large regions of interest or numbers of replicates to give reasonable estimates, and its implementation has been simplified by using confocal microscopy in conjunction with fluorescent inks to record the distribution of nuclei in the casts. Preliminary data showed good agreement with the macroscopic dimensions of the casts, but further work is required to optimise the casting technique itself, so that the data are more relevant to *in vivo* conditions, and to improve retention of endothelium during the casting process. Ultimately, the method could be used to examine areas around arterial branches and hence to support or refute the hypothesis that the development of atherosclerosis is influenced by mural strain. Furthermore, the method may be applicable to other tissues where markers of architecture are redistributed under applied stresses.

## Supplementary Material

**Electronic supplementary material** The online version of this article (https://doi.org/10.1007/s11340-020-00655-9) contains supplementary material, which is available to authorized users.

EMS109768_Sup_Materials
