# Supplementary material for: Estimating Arterial Cyclic Strain from the Spacing of Endothelial Nuclei
Source: Exp Mech. Author manuscript; Available in PMC 2021 Jan 27. (PMC7116634; doi:10.1007/s11340-020-00655-9)
Supplement: EMS109768_Sup_Materials [file EMS109768-supplement-EMS109768_Sup_Materials.docx]

|  |  |  |  |  | NN analysis | | Retro-deformation & NN analysis | |
| --- | --- | --- | --- | --- | --- | --- | --- | --- |
| Rabbit | Sample | $n$ | $\lambda_{r,1}$ | $\phi$(°) | $\overline{\left\vert w_{x} \right\vert}$ | $\overline{\left\vert w_{y} \right\vert}$ | $\overline{\left\vert v_{x} \right\vert}$ | $\overline{\left\vert v_{x} \right\vert}$ |
| A | 1 | 429 | 2.55 | 176.5 | 11.65 | 8.82 | 7.48 | 16.22 |
| 9 weeks | 2 | 429 | 2.88 | 174.0 | 11.81 | 8.58 | 7.23 | 17.23 |
| 1.68 kg | 3 | 399 | 3.18 | 180.0 | 12.17 | 8.92 | 7.69 | 17.80 |
|  | 4 | 442 | 2.70 | 179.5 | 11.93 | 8.67 | 7.35 | 16.19 |
|  | 5 | 404 | 3.45 | 178.0 | 13.26 | 9.40 | 6.51 | 23.65 |
| B | 1 | 421 | 3.33 | 178.5 | 12.16 | 9.14 | 6.58 | 20.12 |
| 9 ½ weeks | 2 | 387 | 2.45 | 176.5 | 12.77 | 9.43 | 8.19 | 16.87 |
| 1.84 kg | 3 | 425 | 3.15 | 176.5 | 12.49 | 8.45 | 6.69 | 19.96 |
|  | 4 | 391 | 3.13 | 174.5 | 12.99 | 8.85 | 7.57 | 20.22 |
| C | 1 | 501 | 3.15 | 163.5 | 11.34 | 8.73 | 7.96 | 19.78 |
| 11 weeks | 2 | 453 | 2.15 | 156.0 | 11.30 | 9.45 | 9.76 | 14.05 |
| 1.83 kg | 3 | 545 | 2.58 | 164.0 | 10.74 | 8.52 | 7.56 | 15.72 |
| Mean |  | 435.50 | 2.89 | 173.1 | 12.05 | 8.91 | 7.55 | 18.15 |

**Table s1** Strain analysis results summary for EC nuclear point patterns from the 90 mmHg group

|  |  |  |  |  | NN analysis | | Retro-deformation & NN analysis | |
| --- | --- | --- | --- | --- | --- | --- | --- | --- |
| Rabbit | Sample | $n$ | $\lambda_{r,1}$ | $\phi$(°) | $\overline{\left\vert w_{x}^{'} \right\vert}$ | $\overline{\left\vert w_{y}^{'} \right\vert}$ | $\overline{\left\vert v_{x}^{'} \right\vert}$ | $\overline{\left\vert v_{y}^{'} \right\vert}$ |
| A | 1 | 286 | 3.58 | 168.0 | 14.23 | 10.45 | 10.90 | 22.73 |
| 9 ½ weeks | 2 | 238 | 2.95 | 167.0 | 15.92 | 11.82 | 10.89 | 23.08 |
| 1.69 kg | 3 | 293 | 2.28 | 172.5 | 14.62 | 10.71 | 11.04 | 17.22 |
|  | 4 | 322 | 2.30 | 170.0 | 13.93 | 10.53 | 10.57 | 16.36 |
|  | 5 | 283 | 3.53 | 172.0 | 14.90 | 11.72 | 9.67 | 25.46 |
|  | 6 | 305 | 2.93 | 166.0 | 14.50 | 10.90 | 11.20 | 21.54 |
| B | 1 | 317 | 3.55 | 161.0 | 13.27 | 10.57 | 11.40 | 23.24 |
| 9 weeks | 2 | 325 | 3.08 | 178.5 | 13.15 | 9.05 | 9.34 | 17.03 |
| 1.72 kg | 3 | 353 | 3.50 | 177.5 | 12.96 | 10.56 | 8.28 | 21.28 |
| C | 1 | 339 | 1.65 | 172.0 | 12.27 | 10.18 | 10.82 | 12.08 |
| 9 weeks | 2 | 333 | 2.48 | 175.5 | 12.93 | 10.04 | 9.88 | 14.86 |
| 1.96 kg | 3 | 341 | 3.13 | 173.5 | 12.80 | 9.60 | 8.65 | 18.78 |
| Mean |  | 311.25 | 2.75 | 171.1 | 13.79 | 10.51 | 10.22 | 19.47 |

**Table s2** Strain analysis results summary for EC nuclear point patterns from the 120 mmHg group

|  | Rabbit | Intercostal pair | Axial spacing (mm) | | Max insc. sph. radius (mm) |
| --- | --- | --- | --- | --- | --- |
|  |  |  | L | R |  |
| 120 mmHg | A | 1-2 | 8.616 | 8.546 | 2.559 |
|  | 9 ½ weeks | 2-3 | 8.970 | 8.136 | 2.591 |
|  | 1.69 kg | 3-4 | 8.585 | 9.179 | 2.572 |
|  |  | 4-5 | 9.099 | 9.405 | 2.495 |
|  |  |  |  |  |  |
|  | B | 1-2 | 9.072 | 9.741 | 2.783 |
|  | 9 weeks | 2-3 | 9.713 | 9.314 | 2.782 |
|  | 1.72 kg | 3-4 | 10.080 | 8.611 | 2.732 |
|  |  | 4-5 | 8.964 | 10.515 | 2.515 |
|  |  |  |  |  |  |
|  | C | 1-2 | 7.07 | 8.79 | 2.76 |
|  | 9 weeks | 2-3 | 8.32 | 8.17 | 2.77 |
|  | 1.96 kg | 3-4 | 8.77 | 8.56 | 2.60 |
|  |  | 4-5 | 9.65 | 9.81 | 2.61 |
|  | Mean |  | 8.909 | 9.065 | 2.647 |
| *Continued…* |  |  |  |  |  |
| 90 mmHg | D | 1-2 | 7.548 | 8.323 | 1.85 |
|  | 9 weeks | 2-3 | 7.92 | 7.445 | 1.843 |
|  | 1.68 kg | 3-4 | 8.93 | 9.872 | 1.839 |
|  |  | 4-5 | 10.43 | 11.023 | 1.798 |
|  |  |  |  |  |  |
|  | E | 1-2 | 8.851 | 8.359 | 1.87 |
|  | 9 ½ weeks | 2-3 | 9.201 | 9.104 | 1.926 |
|  | 1.84 kg | 3-4 | 9.409 | 10.428 | 1.97 |
|  |  | 4-5 | 10.886 | 10.285 | 1.872 |
|  |  |  |  |  |  |
|  | F | 1-2 | 7.881 | 6.928 | 1.69 |
|  | 11 weeks | 2-3 | 6.988 | 8.257 | 1.628 |
|  | 1.83 kg | 3-4 | 8.468 | 7.582 | 1.598 |
|  |  | 4-5 | 9.318 | 8.533 | 1.67 |
|  |  |  |  |  |  |
|  | Mean |  | 8.819 | 8.845 | 1.796 |
|  |  |  |  |  |  |
|  | Stretch |  | 1.010 | 1.025 | 1.474 |
|  | *p* value |  | 0.868 | 0.722 | 0.73x 10^-3^ |
|  |  |  |  |  |  |

**Table s3** Axial distance between intercostal arteries on the left and right sides, and aortic radius determined from the maximum radius of inscribed spheres, at 120 and 90 mmHg

**
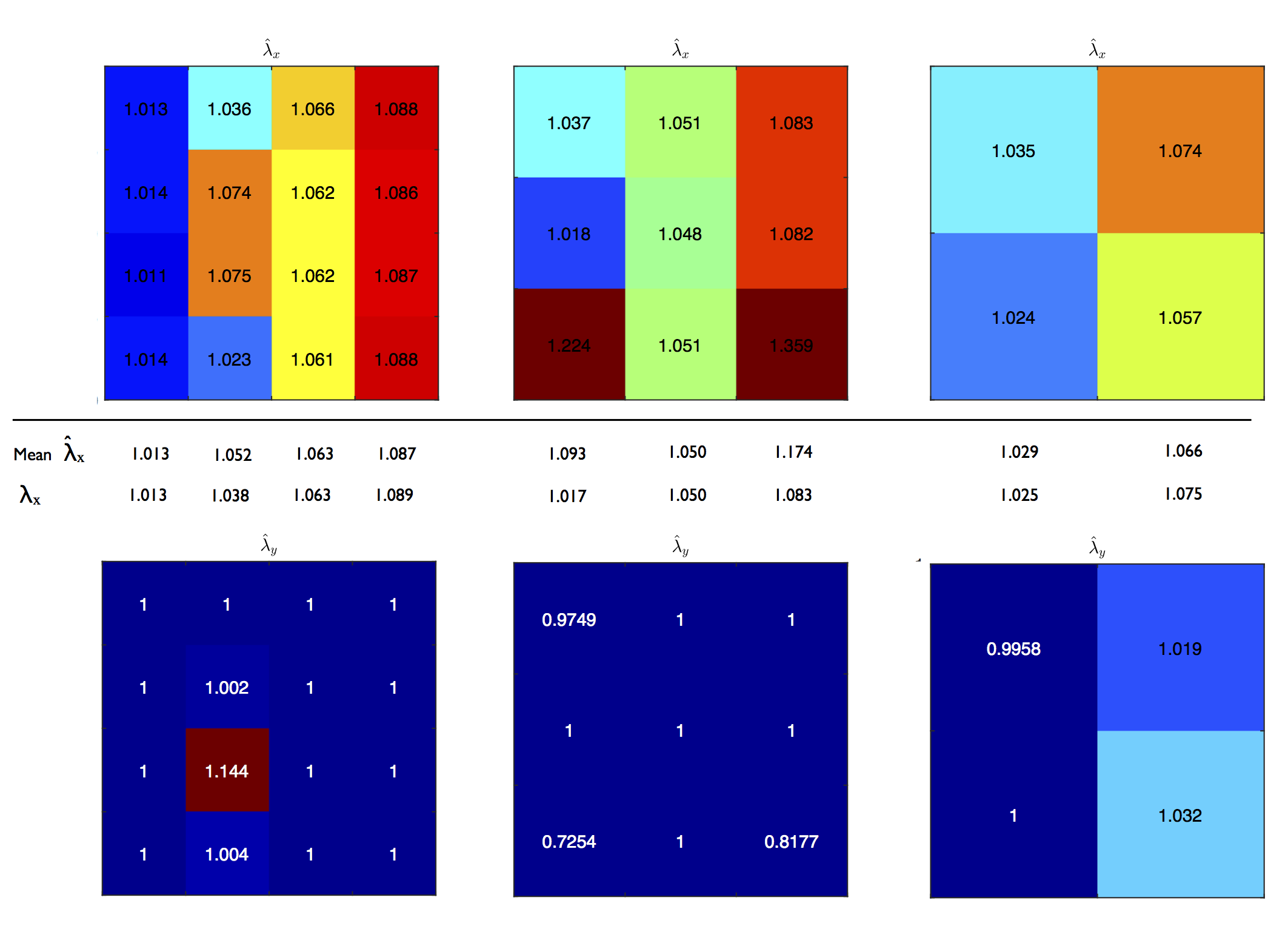
**

**Figure s4** EC nuclear point pattern subjected to a linear deformation gradient *x*’ = 0.05*x*^2^+*x*. Each small box represents a square ROI in the reference configuration. Left column: 125x125 μm. Middle column: 167x167 μm. Right column: 250x250 μm. Values are stretch estimates computed with combined retro-deformation and NN analysis. Top row: $\hat{\lambda}_{x}$, column averages and expected values given below. Bottom row: $\hat{\lambda}_{y}$, expected value 1.

**
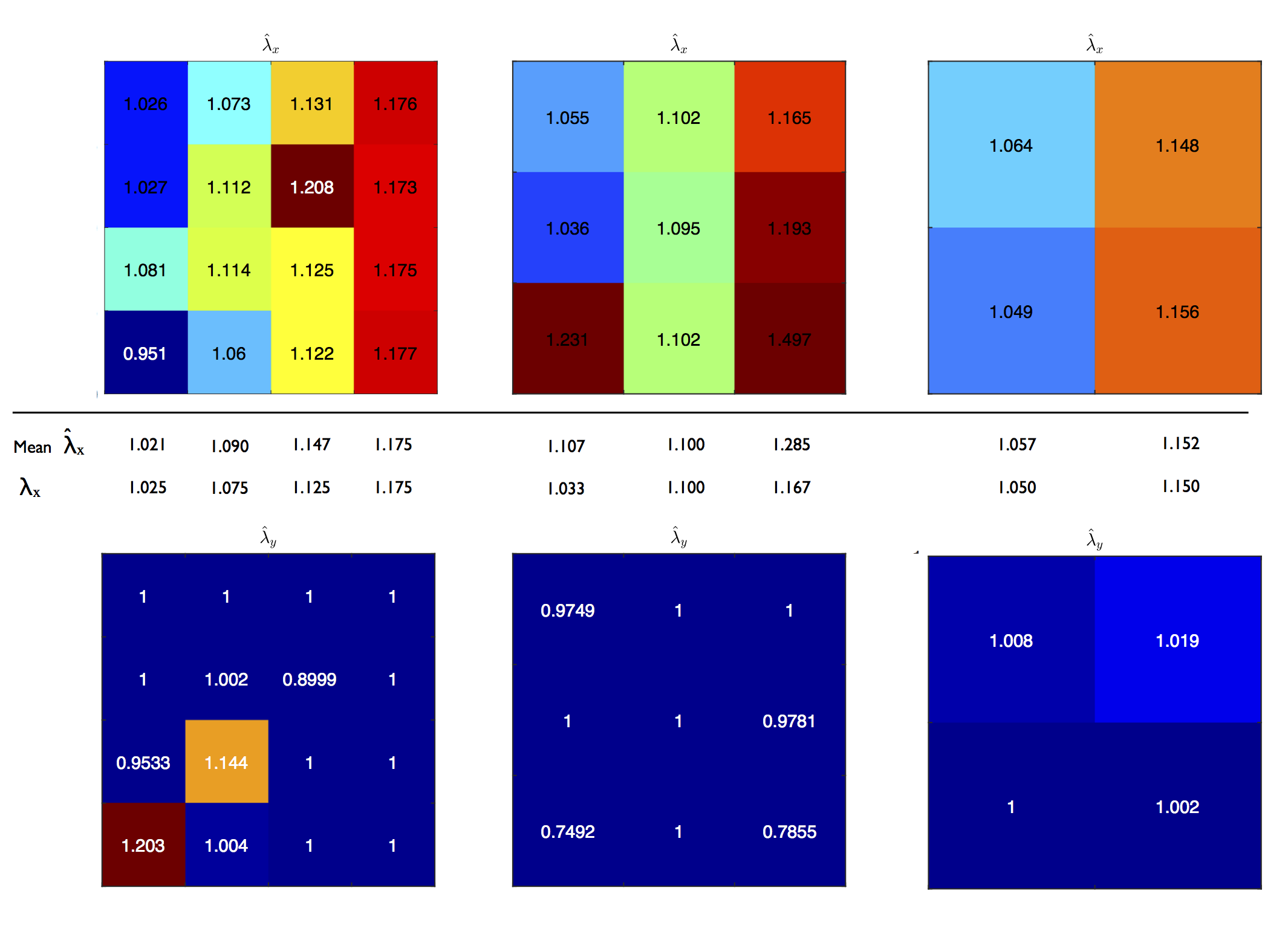
**

**Figure s5** EC nuclear point pattern subjected to a linear deformation gradient *x*’ = 0.1*x*^2^+*x*. Each small box represents a square ROI in the reference configuration. Left column: 125x125 μm. Middle column: 167x167 μm. Right column: 250x250 μm. Values are stretch estimates computed with combined retro-deformation and NN analysis. Top row: $\hat{\lambda}_{x}$, column averages and expected values given below. Bottom row: $\hat{\lambda}_{y}$, expected value 1.
